# Reorganization

**Published:** 1865

**Authors:** 


### REORGANIZATION.

The Mad River Dental Association, after a suspension of operations, has recently reorganized with better indications and prospects than at any other time. Two meetings have recently been held, at both of which much interest was manifested, and henceforth, we doubt not, it will progress with a steady vigor and usefulness. We hope that all within the sphere of its influence will avail themselves of the advantages derivable therefrom.

